# Estimates of non-communicable disease expenditure by disease phase, sex, and age group for all OECD countries

**DOI:** 10.1186/s12963-025-00418-5

**Published:** 2025-10-08

**Authors:** Samantha Grimshaw, Emily Bourke, Tony Blakely

**Affiliations:** Population Interventions Unit, Centre for Epidemiology and Biostatistics, Melbourne School of Population and Global Health, University of Melbourne, Carlton, VIC Australia

**Keywords:** Disease expenditure, Health expenditure, OECD countries, Non-communicable diseases

## Abstract

**Background:**

NCD expenditure estimates are necessary to estimate future health system expenditure trajectories for different prevention and treatment policies. However, no dataset of comparable estimates exists across OECD countries. This study generates disease expenditure estimates for all 38 OECD member countries in 2019, for 80 major NCDs by disease phase, sex, and age group.

**Methods:**

Australian health expenditure (per person) by sex and age group was disaggregated by disease phase (first year of diagnosis, last year of life if dying of disease, otherwise prevalent) using Global Burden of Disease (GBD) data and New Zealand estimates of relative expenditure ratios by phase. These estimates were applied to GBD estimated case numbers in each OECD country and scaled to each country’s total health system expenditure to estimate expenditure by NCDs in 2019. OECD purchasing power parities were used to adjust estimates to United States (US) dollars for cross-country comparability. Comparisons were made to pre-existing disease expenditure estimates for Norway, Switzerland, and the US.

**Results:**

Average NCD expenditure across OECD countries was US$207 million per 100,000 population. Pooled across countries, musculoskeletal disorders had the highest proportion of total health expenditure (17.4%), followed by cancer (9.4%), and cardiovascular diseases (CVD) (9.1%). Within diseases, the percentage of expenditure was higher for females for musculoskeletal disorders (56.1%), mental and substance use disorders (55.8%), and neurological conditions (54.8%). For males, it was kidney and urinary diseases (63.8%), cancer (58.3%), and CVD (50.7%). First year of diagnosis represented on average 36.8% of total NCD expenditure, while last year of life expenditure accounted for 2.6%.

While there were similarities between our expenditure estimates and pre-existing country-specific estimates for Norway, Switzerland and the US, notable differences were observed for musculoskeletal disorders, cancer, and mental and substance use disorders.

**Conclusions:**

Our estimates represent a starting point for a cross-national dataset of disease-specific expenditure that can be used to forecast future expenditure and potential health system cost savings of preventive and treatment policies. We recommend evolving our paper’s methods to include multiple country-level studies as inputs – augmented by covariates (e.g. GDP, public/private split) to better predict disease expenditure.

**Supplementary Information:**

The online version contains supplementary material available at 10.1186/s12963-025-00418-5.

## Background

Non-communicable diseases (NCDs) are the leading cause of death and disability worldwide, responsible for nearly two-thirds of all yearly deaths and 81% of years lived with a disability [1, 2]. Across the 38 Organisation for Economic Co-operation and Development (OECD) member countries (34 high-income and 4 upper-middle income economies), more than 10.4 million people died from NCDs in 2021–77% of all deaths. Cardiovascular disease (CVD) and cancer accounted for most of this burden (35% and 31% of NCD deaths respectively), followed by neurological disorders (11%), diabetes and kidney diseases (8%), and chronic respiratory diseases (7%) [2]. These conditions also represent a significant disability burden, contributing a total of 208.6 million disability-adjusted life years (DALYs) – equivalent to 46% of all DALYs across member countries [2]. But the impact of NCDs goes well beyond mortality and morbidity statistics.

NCDs also impose a large financial burden on households, health systems and economies that oftentimes require expensive treatment regimens and specialised patient care. Cost of illness studies vary enormously in their perspectives and methods, but as one example the World Economic Forum and Harvard School of Public Health (2011) made a forecast of US$30 trillion lost from NCDs worldwide from 2011 to 2030 – of which roughly half will be due to direct health care costs and the other half to indirect productivity loss and other social costs [3]. A systematic review of cost of illness studies found that CVD accounts for the highest proportion of total health expenditure in most countries, ranging from 12 to 16.5% - but also found marked variation in health system expenditure by NCDs [4] – raising issues of what variation is real as opposed to due to methodological differences in study design and methods.

Ideally, the world would have estimates of disease expenditure for every country that were both comparable in methodology used to generate estimates (e.g. classification of disease; inclusion of inpatient, outpatient, primary care, long-term and aged care) and ‘correct’ to each country’s context and health system configuration (e.g. accounting for differences in pharmaceutical use and costs by country). Such ideal data would have at least three uses: first, to assist prioritising of preventive and treatment interventions informed by impact on future health system expenditure in addition to impact on health gain (i.e. as the ‘cost-offset’ part of net health system costs in cost effectiveness studies); second, to assist planning of future health system budgets in the face of aging populations; and third, to harness cross-national comparisons to inform policy development. Put another way, we propose a vision for disease expenditure data by country that parallels disease epidemiology data as per the global burden of disease (GBD). But this ideal is a long way off, requiring standardisation of methods and data across countries. The purpose of this current paper is to make one step to this ‘ideal’ for OECD countries, by assuming the relative differences in disease expenditure observed in Australasia apply to other OECD countries – employing common GBD disease classifications and country-specific GBD estimates of disease prevalence. Several studies have estimated the economic burden of specific NCD groups, such as CVD and diabetes, and their associated conditions [5, 6]. But, only a handful of countries have comprehensively disaggregated total health expenditure at the country-level across many or all diseases, including Australia, New Zealand (NZ), Norway, Switzerland, and the United States of America (USA) [7–11]. The OECD were the first to release preliminary disease expenditure estimates based on comparable health expenditure data under the System of Health Accounts (SHA) 2011 framework [12]. Data availability was limited – less than half of all OECD countries had produced expenditure data disaggregated by disease groups, and differences in data coverage led to large unallocated expenditure [12]. However, the analysis showed that most allocated health expenditure was on NCDs [12]. Women accounted for more than half of total spending due to higher expenditure on mental health and musculoskeletal conditions, while men had more hospital spending due to their increased expenditure on CVD, injuries, and mental health conditions [12].

Our study builds on previous work by generating comparable NCD expenditure estimates by disease phase, sex and age group for all 38 OECD member countries (all ages) – filling a critical information gap in global health metrics. OECD member countries were chosen for this study as we assumed that health expenditure in Australia and NZ would be comparable to other member countries due to similarities in economies (most being high-income economies) and disease burden patterns (i.e. NCDs account for most of the disease burden in high-income countries).

## Methods

**Fig. 1:**
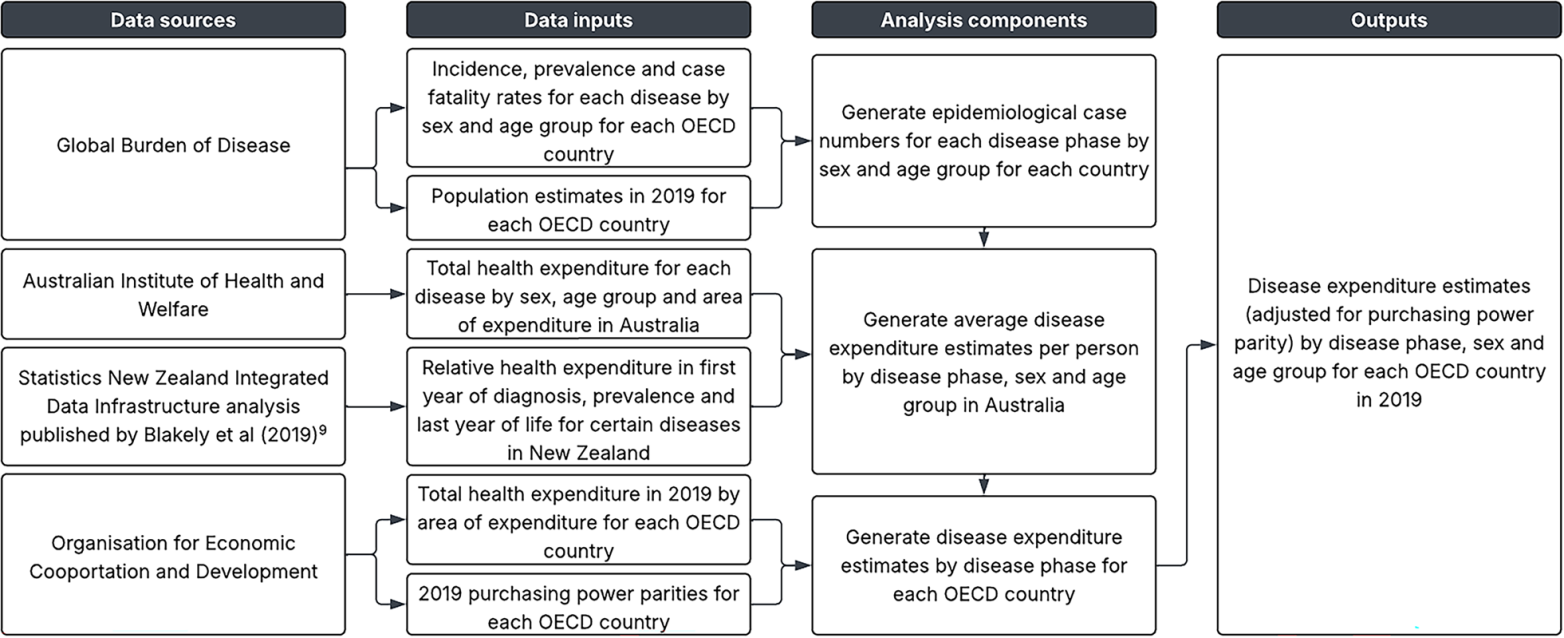
Overview of the study’s data sources, data inputs, analysis components, and outputs

Our methodology used comprehensive Australasian health expenditure estimates as the starting point: Australian disease expenditure by sex, age group and area of expenditure from the Australian Institute of Health and Welfare (AIHW), and relative health expenditure by disease phase from Blakely et al.’s analysis of Statistics New Zealand Integrated Data Infrastructure (IDI) data (see Fig. 1) [8, 13]. These data were combined with estimated disease numbers from the Global Burden of Disease (GBD) for each OECD country to estimate ‘naïve’ NCD expenditure with the Australian cost base [14]. These naïve estimates were then uniformly scaled within each OECD country to ensure the values across all diseases summed to each country’s total health system expenditure as given by OECD national health accounts [15].

All statistical analyses were performed using R (version 4.1.3). NCD expenditure estimates are reported as 2019 US$ using OECD purchasing power parties (PPPs) [16].

### Data sources

Data inputs used to generate our NCD expenditure estimates were derived from four data sources: GBD, AIHW, a Statistics New Zealand IDI analysis and the OECD.

## Global Burden of Disease (GBD)

GBD’s 2019 study quantified health loss across 369 diseases and injuries, with methods described in detail elsewhere [17]. The data extracted from the GBD for this study were the disease prevalence, the number of new incident disease cases, and disease-specific deaths in 2019. Our study included 80 Level 3 NCD causes (see Table S1 for more detail). GBD 2019 prevalence results use point prevalence, which we have assumed to be mid-year point prevalence.

## Australian Institute of Health and Welfare (AIHW)

The AIHWs *Disease expenditure in Australia 2018-19* report used a range of modelling techniques to allocate health expenditure on 219 conditions across the Australian population from 1 July 2018 to 30 June 2019 [13]. Disease expenditure estimates were generated using top-down and bottom-up approaches, which involved estimating Australia’s total health expenditure and then allocating it across the conditions based on available service data from various data sources, including health care databases and surveys [18]. Approximately 73% of recurrent expenditure was able to be attributed to the relevant conditions [13].

Areas of expenditure included in the analysis were hospital services, referred and unreferred medical services, benefit paid pharmaceuticals, dental, and other health practitioners and community health services [13]. Areas of expenditure that remained largely unallocated were over-the-counter pharmaceuticals, other health practitioners, community health, public health, and research [18].

Our study used AIHW’s disease expenditure estimates (by sex and age group) for 91 conditions (see Table S1 for more detail).

## Statistics New Zealand Integrated Data Infrastructure (IDI) analysis

Blakely et al.’s [8] analysis used statistical methods to disaggregate publicly funded health expenditure disease in NZ from 1 July 2007 to 30 June 2014 [8]. Areas of expenditure included in the analysis were hospitalisation, outpatient, pharmaceutical, laboratory testing, and primary care [8]. A case definition algorithm was used to identify anyone with any of 14 NCDs across various national health care datasets (lung cancer, colorectal cancer, breast cancer, prostate cancer, other cancer, ischaemic heart disease (IHD), stroke, other CVD, type 2 diabetes mellitus, chronic lung disease, chronic kidney disease, chronic liver disease, neurological conditions, and musculoskeletal conditions) [8]. The output from this NZ study used in the current study as the relative differences in expenditure by disease phase (first year of diagnosis, last year of diagnosis if dying of the disease, otherwise prevalent with the disease).

## The Organisation for Economic Cooperation and Development (OECD) System of Health Accounts (SHA)

The OECD have collaborated with the WHO and European Commission to establish a global framework for producing health expenditure accounts – the SHA 2011 [19]. This framework tracks health care expenditure, mainly functions of health care (referred to as ‘area of expenditure’ in our study), health care provision, and financing schemes, across 38 OECD member countries and some non-member countries [15]. To generate expenditure data for these countries, governments fill out and submit the Joint Health Accounts Questionnaire based on the SHA 2011 framework [19].

For our study, we have used the OECD’s 2019 data on total health expenditure by area of expenditure for each member country, as we assumed that this health care expenditure could be allocated across diseases [15].

### Study populations

Each OECD member country in 2019 were categorised into sex by 19 age groups (< 1 year, 1–4 years, 5–9 years, …, 85 + years) using the GBD 2019 population estimates (see Table S2 for more detail) [20]. 

## Concordance of Australian conditions to GBD causes

There were 91 AIHW conditions that corresponded to 80 level 3 NCD causes from the GBD 2019 study (referred to as ‘GBD causes’ henceforth) across 10 ‘disease groups’ (see Table S1). Inflammatory heart disease (one of the AIHW conditions) corresponded with two GBD causes: ‘cardiomyopathy and myocarditis’, and ‘endocarditis’. Therefore, inflammatory heart disease’s expenditure was disaggregated across the two GBD causes based on the number of prevalent cases for each cause.

In each of the 10 disease groups, there were AIHW conditions that did not have an exactly corresponding GBD cause. Therefore, 10 additional groupings were used that corresponded to the remaining AIHW conditions (e.g. ‘all other cancer conditions’ and ‘all other CVD conditions’).

Finally, an ‘all other’ category capture remaining AIHW health expenditure, i.e. injuries; communicable, maternal, neonatal and nutritional diseases; and minor NCDs (e.g. congenital birth defects, oral disorders).

## Disease phase

Each of the 80 GBD causes were assigned a corresponding relative disease phase costing ratio from Blakely et al.’s 2019 analysis (see Table S1) [8]. For those GBD causes that could not be matched with one of the 14 ratios from the analysis, they were assigned either a ‘flat’ ratio (i.e. a 1 : 1 : 1 ratio; for diseases whose treatment is expected to be similar across phase) or an ‘average’ ratio (i.e. a 2.40 : 1: 2.72 ratio; remaining diseases) that was the average of all 14 ratios from the analysis) [8].

### OECD total health expenditure

Three areas of expenditure (governance and health system and financing administration, long-term care, and preventative care) were excluded from the OECD’s total health expenditure estimates for each member country [18]. This was due to the AIHW being unable to allocate these areas of expenditure by disease. Colombia, Israel, NZ, and Turkey did not have expenditure estimates for each area of expenditure, only a total health expenditure estimate. 81% of health care expenditure across all other OECD countries was due to areas excluding the three above areas of expenditure; we therefore scaled Colombia, Israel, NZ, and Turkey’s total health expenditure by 81% (see Figure S1).

### Generating epidemiological case numbers for each disease phase by sex and age group for each country

For each disease, sex, and age group combination, incidence, prevalence and mortality risks from the GBD for 2019 were multiplied by population estimates to generate the number of cases for each OECD country. As the GBD uses mid-year point prevalence, the finalised number of period (annual) prevalent cases was calculated by subtracting off half the number of incident cases and half the number of decedents.

The 10 additional disease groups (e.g. ‘all other cancer conditions’, ‘all other CVD conditions’) did not have GBD data. We therefore assigned an estimated prevalence based on relativities in the Australian data, e.g. if ‘other CVD’ prevalence was 15% of all prevalent CVD in a given sex by age group in Australia, we assigned an ‘other CVD’ prevalence in other OECD country of 15/85 multiplied by the sum of GBD estimated prevalences for non-other CVD ‘GBD causes’. Likewise, a prevalence of ‘all other’ disease (i.e. non-NCD) was estimated for each OECD country.

### Generating disease expenditure estimates per person by sex, age group and phase for Australia

The expenditure (in US$) per prevalent phase case in Australia of disease (d), within each sex (s) and age group (a) was:

$$\:{Exp\:prev\:cases}_{Aus,\:dsa}=\:\:\frac{{Exp\:all\:cases}_{Aus,\:dsa}}{\sum\:_{phase}\left({ratio}_{phase,\:Aus,\:dsa}\times\:{N}_{phase,Aus,\:dsa}\right)}$$

where ‘exp all cases’ is the AIHW estimate of disease expenditure (i.e. not disaggregated by phase), ‘ratio’ is the relative ratio of expenditure for incident or last year of life cases compared to otherwise prevalent cases from NZ, and N is the number of disease cases in Australia in 2019 by phase estimated from GBD data (above). The expenditure per incident and last year of life (if dying of that disease) was the above prevalent estimate multiplied by its phase ratio.

### Generating disease expenditure estimates per person by sex, age group and phase for each OECD member country

A naïve (i.e. using the Australian cost-base with no PPP or other adjustment) initial estimate of total disease expenditure in each OECD country (c) was estimated:

$$\:\sum_{phase,dsa}\left({N}_{phase,c,\:dsa}\times\:\:{Exp}_{phase,Aus,dsa}\right)$$

where $\:{N}_{phase,c,\:dsa}$ is the number of disease cases by sex, age and phase in each country *c* estimated using GBD data. Then scalars were estimated for each country:

$$\:{Scalar}_{c}=\:\:\frac{{Total\:expenditure\:national\:health\:accounts}_{c}}{\sum\:_{phase,dsa}\left({N}_{phase,c,\:dsa}\times\:\:{Exp}_{phase,Aus,dsa}\right)}$$

The country specific scalars were then multiplied by the Australian disease expenditure per person estimates ($\:{Exp}_{phase,Aus,dsa}$) to generate country specific estimates of expenditure by disease phase, sex and age group (i.e. $\:{Exp}_{phase,c,dsa}$) in US dollars.

### Comparisons with pre-existing health expenditure estimates

We compared our country-level estimates with three separate country studies that used a bottom-up costing of services by diagnosis using similar methods to the AIHW’s disease expenditure study: Norway [9], Switzerland [10] and the USA [11].

## Results

### Health spending on NCDs across OECD member countries

**Fig. 2:**
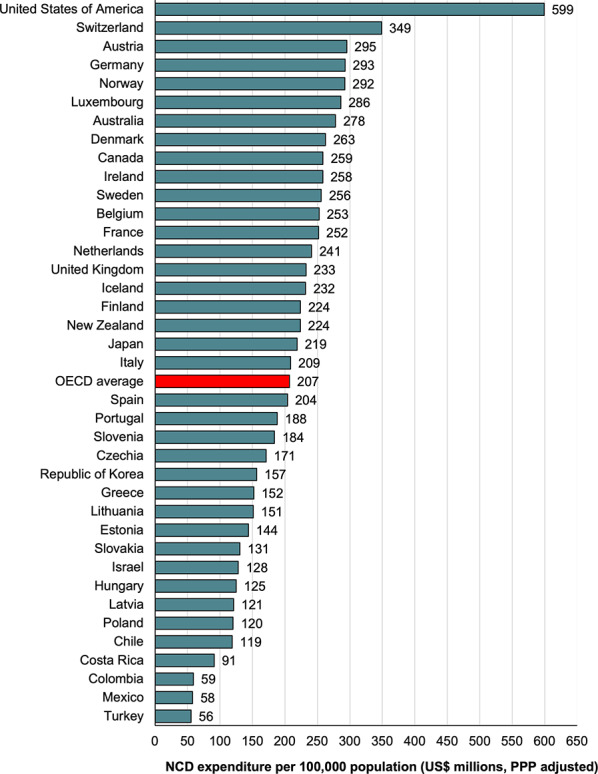
Total disease expenditure per 100,000 population for NCDs across OECD member countries in 2019 (OECD average = US$207 million per 100,000 population)

In 2019, the average health expenditure on NCDs across OECD countries was US$207 million per 100,000 population (see Fig. 2). The United States of America (USA) clearly had the highest per capita spending at US$599 million per 100,000 while Turkey had the lowest expenditure at US$56 million.

**Fig. 3:**
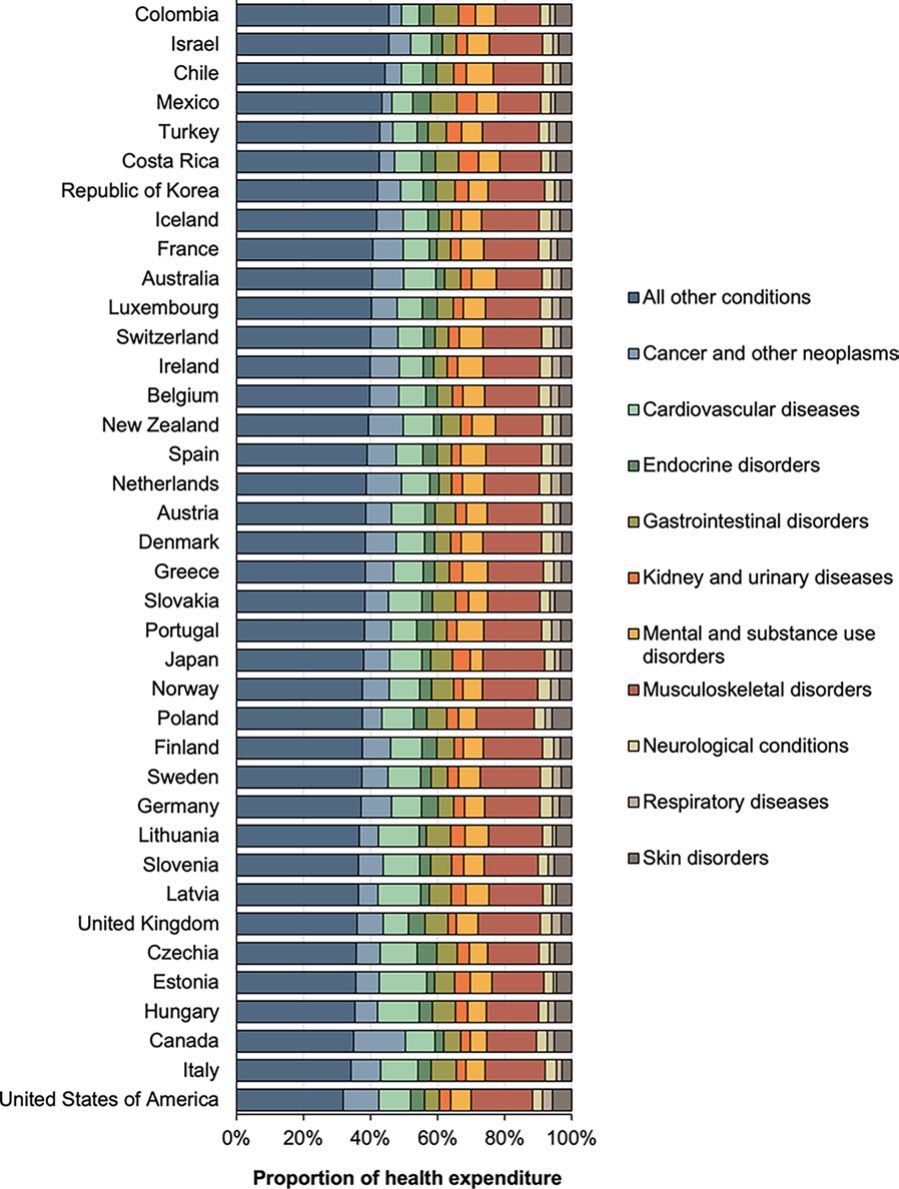
Proportion of total health expenditure by disease groups across OECD member countries in 2019

For all OECD countries pooled, musculoskeletal disorders contributed to the highest proportion of total health expenditure (17.4%), followed by cancer and other neoplasms (9.4%), CVD (9.1%), mental and substance use disorders (6.1%), gastrointestinal disorders (5.0%), skin disorders (4.7%), endocrine disorders (3.9%), kidney and urinary diseases (3.5%), neurological conditions (3.2%), and respiratory diseases (2.5%). The remaining 35.3% of total health expenditure was attributable to other diseases (see Fig. 3 and Supplementary Table 3).

For the top three expenditure conditions, the following percentage expenditure ranges were observed between countries: 18.6% in the United Kingdom to 12.3% in Costa Rica for musculoskeletal disorders; 15.5% in Canada to 3.0% in Mexico for cancer and other neoplasms; and 14.1% in Estonia to 5.4% in Colombia for CVD.

**Fig. 4:**
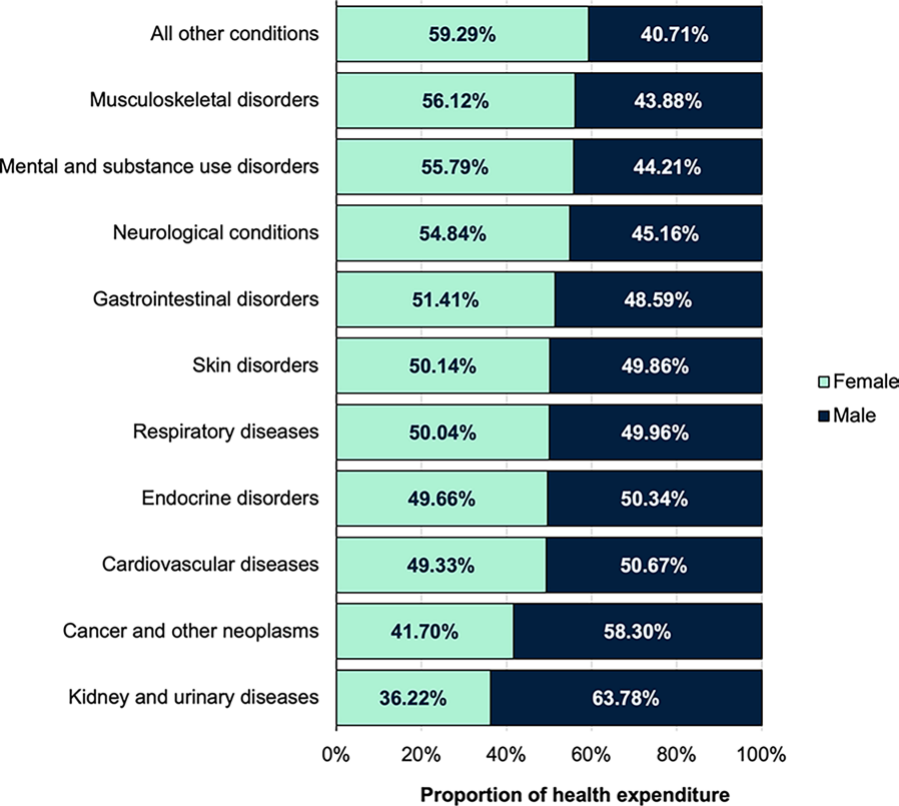
Health expenditure on NCDs by disease group and sex across OECD member countries in 2019; all countries pooled, crude analyses (i.e. no age-standardisation)

Figure 4 shows the proportion of health expenditure on NCDs within sex: females had relatively higher expenditure than males for musculoskeletal disorders (1.28-fold greater or 12.2% points greater), mental and substance use disorders (1.26-fold greater or 11.6% points greater), and neurological conditions (1.21-fold greater or 9.7% points greater) (see Fig. 3). Males had significantly higher expenditure on kidney and urinary diseases (1.76-fold greater or 27.6% points greater), and cancer and other neoplasms (1.40-fold greater or 16.6% points greater).

Across the three disease phases, the majority of total NCD expenditure went to the prevalent phase (at an average across NCDs of 60.7%), followed by the first year of diagnosis (at an average 36.8%), and the last year of life (at 2.6%; noting that this is expenditure attributed to the cause of death, not all expenditure by the health system in the last year of life).

### Comparisons with pre-existing health expenditure estimates

**Fig. 5:**
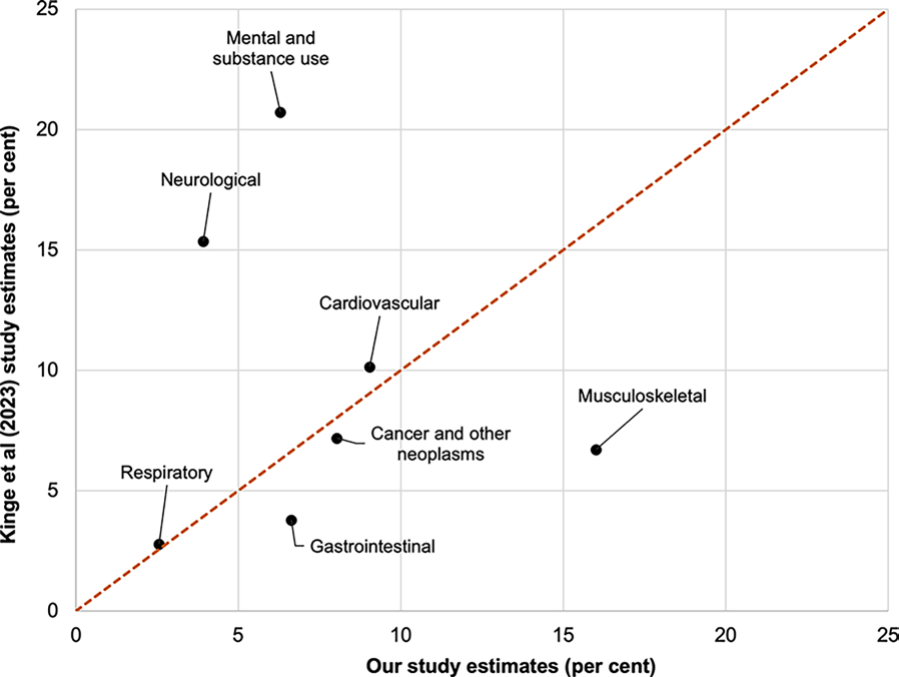
Scatterplot comparing aggregated disease group expenditure estimates from our study (x-axes; percent) with Norway, Kinge et al(2023) [9]

Figure 5 shows the percentage of Norway’s total health expenditure in 2019 across seven aggregated disease groups that were similar between our study and the most recent Norwegian-specific study by Kinge et al. (2023) [9]. There was poor agreement for neurological (3.9% of all health expenditure using our method, compared to 15.4% in the Kinge et al. [9] study), mental and substance use (20.7% and 6.3%) and musculoskeletal (6.7% and 16.0%), but reasonably good agreement for the four other diseases shown.

**Fig. 6:**
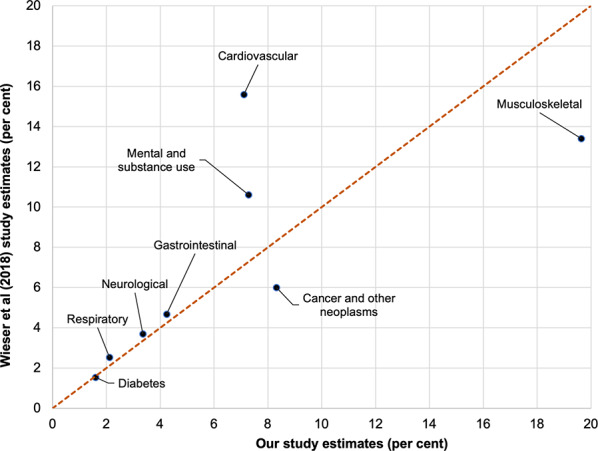
Scatterplot comparing aggregated disease group expenditure estimates from our study (x-axes; percent) with Switzerland,Wieser et al (2018) [10]

Figure 6 shows a similar comparison for Switzerland, for eight comparable disease groupings with Weiser et al. (2018) [10]. There was poor agreement for CVD (7.1% our method, 15.6% Weiser et al. [10]). There was moderate agreement for mental and substance use (7.3% and 10.6%), cancer and other neoplasms (8.3% and 6.0%) and musculoskeletal (19.6% and 13.4%). The remaining four conditions had good agreement.

**Fig. 7:**
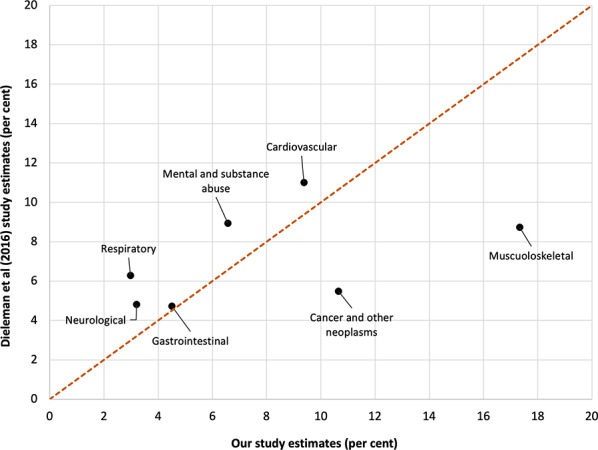
Scatterplot comparing aggregated disease group expenditure estimates from our study (x-axes; percent) with USA,Dieleman et al (2016) [11]

Figure 7 shows a comparison for the USA, for seven comparable groupings with Dieleman et al. (2016) [11]. There was poor agreement for musculoskeletal (17.3% our method, 8.7% Dieleman et al. [11], cancer and other neoplasms (10.6% and 5.5%) and respiratory (3.0% and 6.3%). The other four conditions had moderate to good agreement.

Looking across these three country comparisons, our method consistently estimates greater percentage musculoskeletal disease expenditure than the country-specific studies. Less notable general differences include our method estimating greater cancer and other neoplasm expenditure, and estimating less mental and substance use expenditure.

**Fig. 8:**
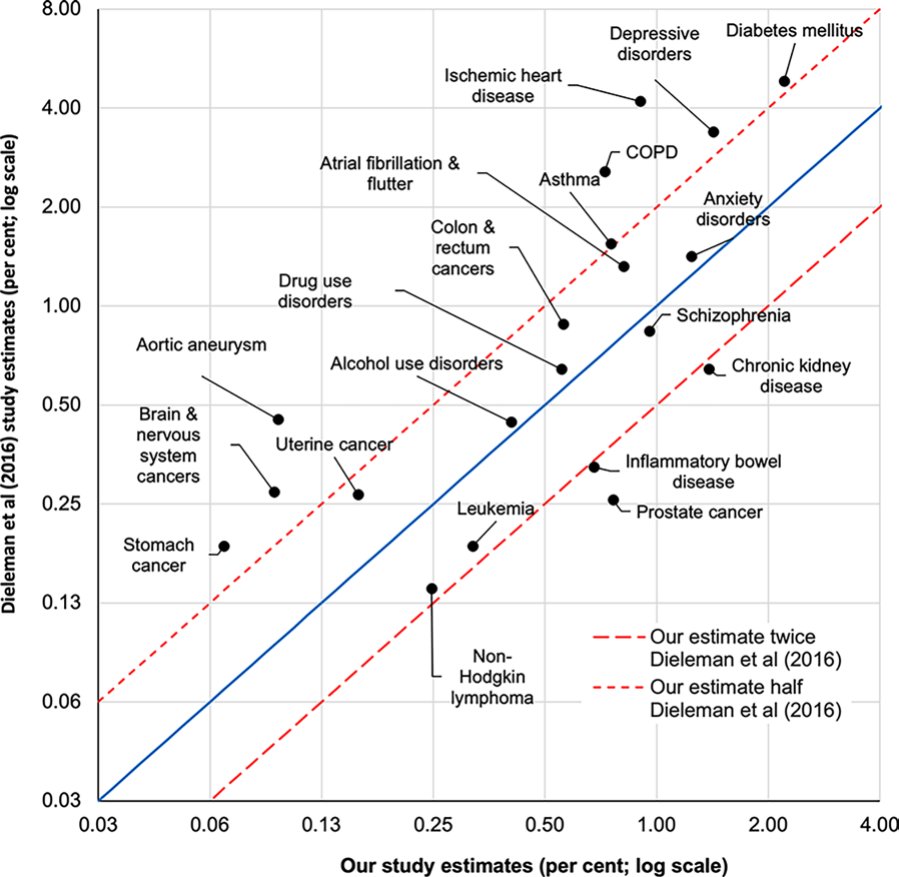
Scatterplot comparing the USA’s proportion of health expenditure on select NCDs using expenditure estimates from this study andDieleman et al (2016) [11]

The Dieleman et al. [11] study also permitted a comparison of 20 more disaggregated conditions (Fig. 8, log scale both axes). For nine of the 20 conditions, our method gave an estimate of the percentage of all expenditure that was within 50–200% of the estimate from Dieleman et al. [11].

## Discussion

Our method estimated an average health expenditure on major NCDs across OECD member countries of US$207 million per 100,000 population. The USA had the highest expenditure at US$599 million per 100,000, and (according to our method) musculoskeletal disorders topped the list of NCDs with the highest proportion of total health expenditure across member countries. After pooling health expenditure on NCDs across countries, females had significantly higher expenditure on musculoskeletal disorders, as well as mental and substance use disorders, and neurological conditions. Males had significantly higher expenditure on kidney and urinary diseases, and cancer and other neoplasms. First year of diagnosis represented on average 36.8% of total NCD expenditure, while last year of life expenditure accounted for 2.6%.

OECD member countries were chosen for this study as we assumed that health expenditure in Australia and NZ would be similar in relative pattern to these countries, given similarities in disease burden patterns (i.e. NCDs are responsible for most of the disease burden in high-income countries). However, for the three country-specific studies that we could compare our method to – Norway, Switzerland, and the USA [9–11] (Figs. 4 and 5) – the agreement on the percentage of expenditure by disease was often poor.

Comparing between expenditure studies is challenging, due to differences in disease group classifications, health care function expenditure areas, and years that the data was based on – as well as ‘actual’ variation in expenditure by disease between Australia (the base of our method) with other countries. In Norway a large amount of expenditure on long term care is considered health care expenditure – whereas in Australia long term care is generally not considered health expenditure and is excluded from disease expenditure estimates. The Norwegian analysis by Kinge et al. (2023) included service data on general practitioners, physiotherapists and chiropractors, day patient, specialised outpatient, inpatient, prescription drugs, home-based long-term care, and nursing homes [9]. The Australian analysis included expenditure on hospital admitted patients, outpatients and emergency departments, government subsidised medical services (GP, specialist, allied health, medical imaging, and pathology), prescription pharmaceuticals, and dental [13]. Given the differences in how health system expenditure is classified between these countries, the Norwegian study had much higher estimated expenditure for dementia than our study.

The USA analysis by Dieleman et al. (2016) included service data on ambulatory care, inpatient care, pharmaceuticals, emergency care, and nursing facility care [11]. While there were differences in service data used in the USA and Australian analyses, differences in medication costs between the two countries would have likely contributed to differences in cost estimates for diseases like diabetes.

The OECD has undertaken initial comparisons of disease expenditure across member countries. While this used different methods, data sources, and had a limited scope, it found that CVD was estimated to be the highest expenditure group, while in our study musculoskeletal was the highest, followed by cancer and other neoplasms then CVD [12]. Females still accounted for higher expenditure on musculoskeletal disorders, and mental and substance use disorders, while males now accounted for higher expenditure on kidney and urinary diseases, and cancer and other neoplasms [12].

### Strengths and limitations

Our study leveraged high quality, comprehensive data from internationally renowned data sources to generate disease expenditure estimates across OECD countries. The GBD is the most comprehensive observational epidemiological study worldwide, while the OECDs online database is the most comprehensive source of comparable statistics on health systems across industrialised countries. The Blakely et al. (2019) analysis utilised linked health data from Statistics NZ, and both that source and the AIHW are known for their comprehensive datasets that capture comorbidity-adjusted costs for each disease and costs to all payers for expenditure areas [8, 13].

There are limitations of this study. Only 73% of recurrent expenditure in Australia was allocated in the AIHWs disease expenditure 2018-19 study [18]. Areas of expenditure not included were over-the-counter pharmaceuticals, other health practitioners, community health, public health, and research remaining unallocated [18]. An assumption of our study is that this missing disease-related expenditure is distributed similarly to the included expenditure (this missing gap being assumed to be another 8% of all health expenditure using the 81% average across OECD countries of national health accounts expenditure on ‘health care functions’ that we assumed equated to the marginal expenditure driven by diseases).

Other key concerns include other OECD countries having different relative expenditure per capita than Australia. For example, the cost and modality (inpatient versus outpatient) of chemotherapy delivery may differ for the same cancer between countries, altering the relative expenditure per capita within cancers. Similarly, the NZ relative costing ratios by disease phase may not be applicable to all OECD countries due to differing medical care and treatment strategies by disease phases, such as approaches to end of life care (expensive treatments versus palliative care).

Australia has a notably higher musculoskeletal disease burden (measured in DALYs by sex and age) than other counties [21]. But estimating musculoskeletal disease expenditure in other OECD countries applied the Australian expenditure per case to each OECD’s prevalence of musculoskeletal disease. Thus the higher musculoskeletal disease expenditure our method estimated in other OECD countries is due to a high expenditure rate per prevalent case in Australia – an area that warrants further research to understand why.

The OECD’s health expenditure accounts may not be capturing all health expenditure within member countries due to within-country limitations on data availability and extent to which data is captured. The GBD estimates of disease prevalence are a function of within country data and smoothing based on similar countries; whether this produces better estimates than ‘just’ using within country disease data is moot. However, for serious bias from GBD estimates to propagate through our method when looking at aggregated diseases (e.g. CVD) would require an over- or under-estimate to apply to most or all the subsidiary diseases in that category.

In this paper, we have not presented uncertainty intervals about estimates – because the AIHW and OECD data do not have uncertainty intervals to propagate through a Monte Carlo analysis. There will be considerable uncertainty about disease expenditure estimates in each OECD country for reasons above through to measurement error in the source data. Such uncertainty is a priority to reduce in future research (next section).

### Research and policy implications

This is the first known study to have produced disease expenditure estimates by disease phase, sex, and age group across many countries. These estimates provide governments and researchers with a tool to cautiously use to quantify the economic and health system impacts of NCDs, identifying priority diseases attributing the most burden to national health expenditure, informing the allocation of health system expenditure, and improving health system efficiency. But our estimates are a ‘first attempt’ at comparable cross-country disease expenditure estimation – improvements are required.

Future research should look further into the reasons for the residual variation between health expenditure across countries, between our study and all three of the Norwegian, Swiss and US studies [9–11]. On the one hand, progress is needed to make country-level expenditure comparisons less prone to methodological and data differences – meaning any remaining differences are ‘true’ and due to (say) differences in pharmaceutical expenditure, patient care pathways, and any number of other factors. On the other hand, as we gain confidence in what the ‘true’ differences in country-level expenditure are, then predictors of such variation should be identified. For example, private versus public expenditure, greater or lesser focus on new technologies, GDP per capita, per capita pharmaceutical expenditure, and such like. We envisage such predictors being used in predictive algorithms (e.g. machine or ensemble learning) that takes what data we can collate between countries and ‘fills in the gaps’ to give better estimates of expenditure by disease in all countries.

## Conclusion

Having access to a comparable set of disease expenditure estimates across OECD countries provides a starting point to understand which diseases contribute the most economic burden, informing decisions on health expenditure allocation and prevention priorities to improve population health and accelerate progress towards achieving international health targets. Nevertheless, the estimates in this study are rudimentary; more research is required to incrementally move to more accurate estimates of expenditure by disease across countries.

## Supplementary Information

Supplementary Material 1.

## Data Availability

Data generated during this study are available from the corresponding author on reasonable request.

## Abbreviations

AIHW: Australian institute of health and welfare
CVD: Cardiovascular disease
DALY: Disability-adjusted life year
GBD: Global burden of disease
GHDx: Global health data exchange
ICD-10: International classification of diseases 10^th^ revision
IDI: Integrated data infrastructure
IHD: Ischaemic heart disease
NCD: Non-communicable disease
NZ: New Zealand
OECD: Organisation for Economic Co-operation and Development
PMSLT: Proportional multistate lifetable
PPP: Purchasing power parity
SDG: Sustainable development goal
SHA: System of health accounts
USA: United States of America
US$: United States dollars
WHO: World Health Organization
