# Supplementary material for: Estimates of non-communicable disease expenditure by disease phase, sex, and age group for all OECD countries
Source: Popul Health Metr. 2025 Oct 8;23:53. doi: 10.1186/s12963-025-00418-5 (PMC12506150; doi:10.1186/s12963-025-00418-5)

**Table S1.** Concordance file for 80 NCDs (80 level 3 GBD causes and 91 AIHW conditions)

| **GBD ID** | **GBD level 3 cause** | **AIHW condition** | **Disease group** | **Disease phase costing ratio** |
| --- | --- | --- | --- | --- |
| 411 | Esophageal cancer | Oesophageal cancer | Neoplasms | Other cancer |
| 414 | Stomach cancer | Stomach cancer | Neoplasms | Other cancer |
| 417 | Liver cancer | Liver cancer | Neoplasms | Other cancer |
| 423 | Larynx cancer | Laryngeal cancer | Neoplasms | Other cancer |
| 426 | Tracheal, bronchus, and lung cancer | Lung cancer | Neoplasms | Lung cancer |
| 429 | Breast cancer | Breast cancer | Neoplasms | Breast cancer |
| 432 | Cervical cancer | Cervical cancer | Neoplasms | Other cancer |
| 435 | Uterine cancer | Uterine cancer | Neoplasms | Other cancer |
| 438 | Prostate cancer | Prostate cancer | Neoplasms | Prostate cancer |
| 441 | Colon and rectum cancer | Bowel cancer | Neoplasms | Colorectal cancer |
| 444 | Lip and oral cavity cancer | Lip and oral cavity cancer | Neoplasms | Other cancer |
| 447 | Nasopharynx cancer | Nasopharyngeal cancer | Neoplasms | Other cancer |
| 453 | Gallbladder and biliary tract cancer | Gallbladder cancer | Neoplasms | Other cancer |
| 456 | Pancreatic cancer | Pancreatic cancer | Neoplasms | Other cancer |
| 459 | Malignant skin melanoma | Melanoma of the skin | Neoplasms | Other cancer |
| 462 | Non-melanoma skin cancer | Non-melanoma skin cancer | Neoplasms | Other cancer |
| 465 | Ovarian cancer | Ovarian cancer | Neoplasms | Other cancer |
| 468 | Testicular cancer | Testicular cancer | Neoplasms | Other cancer |
| 471 | Kidney cancer | Kidney cancer | Neoplasms | Other cancer |
| 474 | Bladder cancer | Bladder cancer | Neoplasms | Other cancer |
| 477 | Brain and central nervous system cancer | Brain and CNS cancer | Neoplasms | Other cancer |
| 480 | Thyroid cancer | Thyroid cancer | Neoplasms | Other cancer |
| 483 | Mesothelioma | Mesothelioma | Neoplasms | Other cancer |
| 484 | Hodgkin lymphoma | Hodgkin lymphoma | Neoplasms | Other cancer |
| 485 | Non-Hodgkin lymphoma | Non-Hodgkin lymphoma | Neoplasms | Other cancer |
| 486 | Multiple myeloma | Myeloma | Neoplasms | Other cancer |
| 487 | Leukemia | Acute lymphoblastic leukaemia (ALL) | Neoplasms | Other cancer |
| 487 | Leukemia | Acute myeloid leukaemia (AML) | Neoplasms | Other cancer |
| 487 | Leukemia | Chronic lymphocytic leukaemia (CLL) | Neoplasms | Other cancer |
| 487 | Leukemia | Chronic myeloid leukaemia (CML) | Neoplasms | Other cancer |
| 487 | Leukemia | Other leukaemias | Neoplasms | Other cancer |
| 492 | Rheumatic heart disease | Rheumatic heart disease (incl. acute rheumatic fever) | Cardiovascular diseases | Other CVD |
| 493 | Ischemic heart disease | Coronary heart disease | Cardiovascular diseases | IHD |
| 494 | Stroke | Stroke | Cardiovascular diseases | Stroke |
| 498 | Hypertensive heart disease | Hypertensive heart disease | Cardiovascular diseases | Other CVD |
| 499 | Cardiomyopathy and myocarditis | Cardiomyopathy | Cardiovascular diseases | Other CVD |
| 499 | Cardiomyopathy and myocarditis | Inflammatory heart disease | Cardiovascular diseases | Other CVD |
| 500 | Atrial fibrillation and flutter | Atrial fibrillation and flutter | Cardiovascular diseases | Other CVD |
| 501 | Aortic aneurysm | Aortic aneurysm | Cardiovascular diseases | Other CVD |
| 502 | Peripheral artery disease | Peripheral vascular disease | Cardiovascular diseases | Other CVD |
| 503 | Endocarditis | Inflammatory heart disease | Cardiovascular diseases | Other CVD |
| 504 | Non-rheumatic valvular heart disease | Non-rheumatic valvular disease | Cardiovascular diseases | Other CVD |
| 509 | Chronic obstructive pulmonary disease | COPD | Chronic respiratory diseases | Chronic lung disease |
| 510 | Pneumoconiosis | Asbestosis | Chronic respiratory diseases | Chronic lung disease |
| 510 | Pneumoconiosis | Other pneumoconiosis | Chronic respiratory diseases | Chronic lung disease |
| 510 | Pneumoconiosis | Silicosis | Chronic respiratory diseases | Chronic lung disease |
| 515 | Asthma | Asthma | Chronic respiratory diseases | Average |
| 516 | Interstitial lung disease and pulmonary sarcoidosis | Interstitial lung disease | Chronic respiratory diseases | Chronic lung disease |
| 516 | Interstitial lung disease and pulmonary sarcoidosis | Sarcoidosis | Chronic respiratory diseases | Chronic lung disease |
| 520 | Other chronic respiratory diseases | Upper respiratory conditions | Chronic respiratory diseases | Chronic lung disease |
| 521 | Cirrhosis and other chronic liver diseases | Chronic liver disease | Digestive diseases | Chronic liver disease |
| 530 | Paralytic ileus and intestinal obstruction | Intestinal obstruction (without hernia) | Digestive diseases | Average |
| 531 | Inguinal, femoral, and abdominal hernia | Abdominal wall hernia | Digestive diseases | Average |
| 532 | Inflammatory bowel disease | Inflammatory bowel disease (IBD) | Digestive diseases | Average |
| 533 | Vascular intestinal disorders | Vascular disorders of intestine | Digestive diseases | Average |
| 534 | Gallbladder and biliary diseases | Gallbladder and bile duct disease | Digestive diseases | Average |
| 535 | Pancreatitis | Pancreatitis | Digestive diseases | Average |
| 541 | Other digestive diseases | Diverticulitis | Digestive diseases | Average |
| 541 | Other digestive diseases | Other gastrointestinal diseases | Digestive diseases | Average |
| 543 | Alzheimer's disease and other dementias | Dementia | Neurological disorders | Neurological |
| 544 | Parkinson's disease | Parkinson disease | Neurological disorders | Neurological |
| 545 | Idiopathic epilepsy | Epilepsy | Neurological disorders | Neurological |
| 546 | Multiple sclerosis | Multiple sclerosis | Neurological disorders | Neurological |
| 554 | Motor neuron disease | Motor neurone disease | Neurological disorders | Neurological |
| 557 | Other neurological disorders | Guillain-Barre Syndrome | Neurological disorders | Neurological |
| 559 | Schizophrenia | Schizophrenia | Mental disorders | Average |
| 560 | Alcohol use disorders | Alcohol use disorders | Substance use disorders | Average |
| 561 | Drug use disorders | Drug use disorders (excl. alcohol) | Substance use disorders | Average |
| 567 | Depressive disorders | Depressive disorders | Mental disorders | Flat |
| 570 | Bipolar disorder | Bipolar affective disorder | Mental disorders | Average |
| 571 | Anxiety disorders | Anxiety disorders | Mental disorders | Flat |
| 572 | Eating disorders | Eating disorders | Mental disorders | Average |
| 575 | Autism spectrum disorders | Autism spectrum disorders | Mental disorders | Flat |
| 578 | Attention-deficit/hyperactivity disorder | Attention deficit hyperactivity disorder | Mental disorders | Flat |
| 579 | Conduct disorder | Conduct disorder | Mental disorders | Flat |
| 582 | Idiopathic developmental intellectual disability | Intellectual disability | Mental disorders | Flat |
| 587 | Diabetes mellitus | Type 1 diabetes | Diabetes and kidney diseases | Type 2 diabetes mellitus |
| 587 | Diabetes mellitus | Type 2 diabetes | Diabetes and kidney diseases | Type 2 diabetes mellitus |
| 589 | Chronic kidney disease | Chronic kidney disease | Diabetes and kidney diseases | Chronic kidney disease |
| 627 | Rheumatoid arthritis | Rheumatoid arthritis | Musculoskeletal disorders | Musculoskeletal |
| 628 | Osteoarthritis | Osteoarthritis | Musculoskeletal disorders | Musculoskeletal |
| 630 | Low back pain | Back pain and problems | Musculoskeletal disorders | Musculoskeletal |
| 631 | Neck pain | Back pain and problems | Musculoskeletal disorders | Musculoskeletal |
| 632 | Gout | Gout | Musculoskeletal disorders | Musculoskeletal |
| 654 | Dermatitis | Dermatitis and eczema | Skin and subcutaneous diseases | Flat |
| 655 | Psoriasis | Psoriasis | Skin and subcutaneous diseases | Flat |
| 661 | Acne vulgaris | Acne | Skin and subcutaneous diseases | Flat |
| 665 | Decubitus ulcer | Ulcers | Skin and subcutaneous diseases | Average |
| 972 | Headache disorders | Migraine | Neurological disorders | Neurological |
| 992 | Upper digestive system diseases | Gastro Oesophageal Reflux Disease (GORD) | Digestive diseases | Average |
| 992 | Upper digestive system diseases | Gastroduodenal disorders | Digestive diseases | Average |

**Table S2.** List of OECD member countries

| **Country** | **Year of accession** | **World Bank income group** |
| --- | --- | --- |
| Australia | 1971 | High |
| Austria | 1961 | High |
| Belgium | 1961 | High |
| Canada | 1961 | High |
| Chile | 2010 | High |
| Colombia | 2020 | Upper middle |
| Costa Rica | 2021 | Upper middle |
| Czechia | 1995 | High |
| Denmark | 1961 | High |
| Estonia | 2010 | High |
| Finland | 1969 | High |
| France | 1961 | High |
| Germany | 1961 | High |
| Greece | 1961 | High |
| Hungary | 1996 | High |
| Iceland | 1961 | High |
| Ireland | 1961 | High |
| Israel | 2010 | High |
| Italy | 1962 | High |
| Japan | 1964 | High |
| Latvia | 2016 | High |
| Lithuania | 2018 | High |
| Luxembourg | 1961 | High |
| Mexico | 1994 | Upper middle |
| Netherlands | 1961 | High |
| New Zealand | 1973 | High |
| Norway | 1961 | High |
| Poland | 1996 | High |
| Portugal | 1961 | High |
| Republic of Korea | 1996 | High |
| Slovak Republic | 2000 | High |
| Slovenia | 2010 | High |
| Spain | 1961 | High |
| Sweden | 1961 | High |
| Switzerland | 1961 | High |
| Turkey | 1961 | Upper middle |
| United Kingdom | 1961 | High |
| United States | 1961 | High |

**Figure S1.** Total health expenditure by areas of expenditure in OECD member countries, 2019


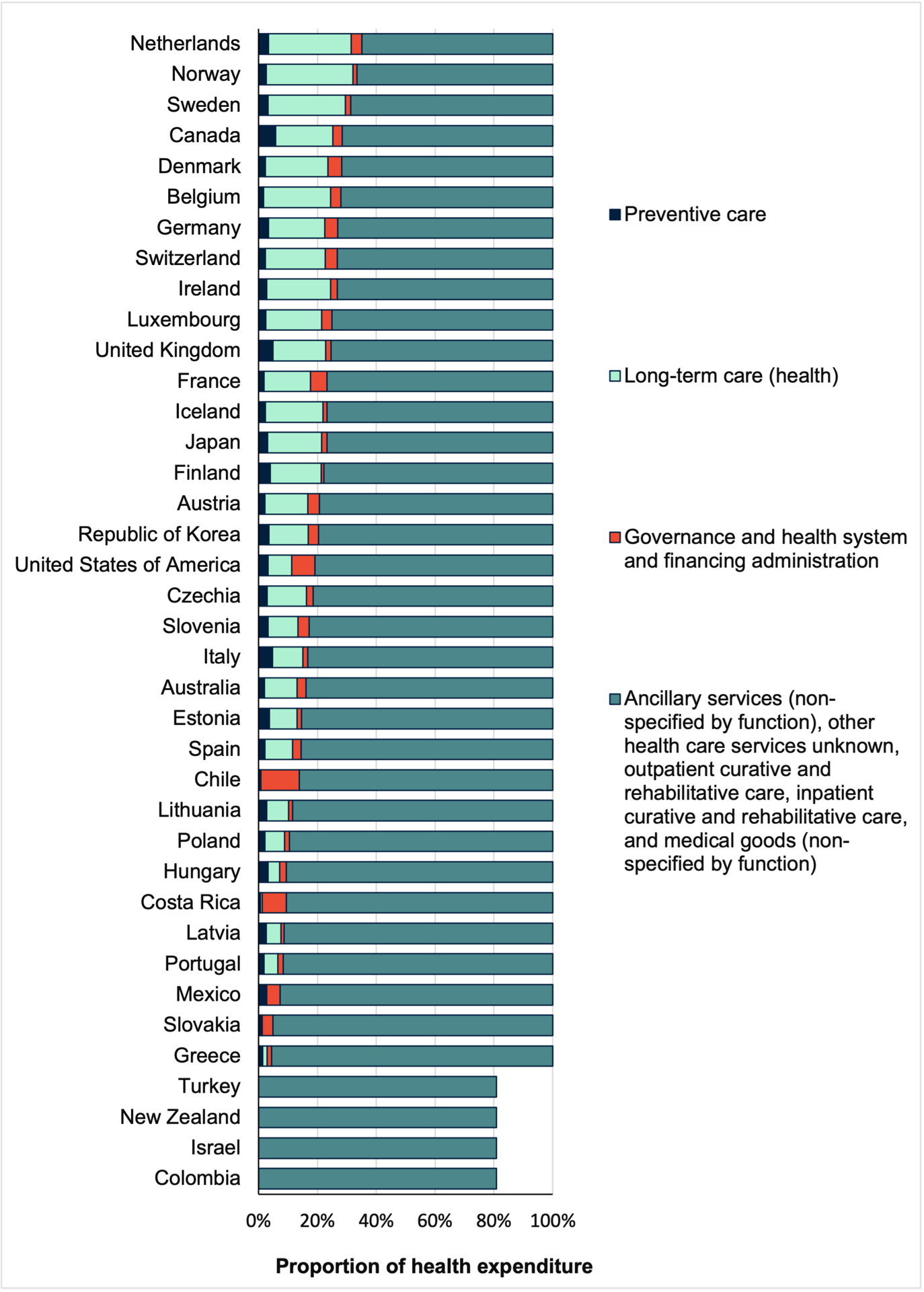

Supplement: Supplementary file 1 — Supplementary Material 1. [file 12963_2025_418_MOESM1_ESM.docx]
